# The Role of Hp-NCL Network in Goal-Directed Routing Information Encoding of Bird: A Review

**DOI:** 10.3390/brainsci10090617

**Published:** 2020-09-07

**Authors:** Mengmeng Li, Zhigang Shang, Kun Zhao, Shuguan Cheng, Hong Wan

**Affiliations:** 1 School of Electrical Engineering, Zhengzhou University, Zhengzhou 450001, China; mengmeng_li@gs.zzu.edu.cn (M.L.); zhaokun2046@126.com (K.Z.); CSG17839194958@163.com (S.C.); 2 Henan Key Laboratory of Brain Science and Brain-Computer Interface Technology, Zhengzhou 450001, China; 3 Institute of Brain and Bioelectronic Information, Zhengzhou University, Zhengzhou 450001, China; 4 Institute of Medical Engineering Technology and Data Mining, Zhengzhou University, Zhengzhou 450001, China

**Keywords:** bird, Hp, NCL, goal-directed, route information encoding

## Abstract

Goal-directed navigation is a crucial behavior for the survival of animals, especially for the birds having extraordinary spatial navigation ability. In the studies of the neural mechanism of the goal-directed behavior, especially involving the information encoding mechanism of the route, the hippocampus (Hp) and nidopallium caudalle (NCL) of the avian brain are the famous regions that play important roles. Therefore, they have been widely concerned and a series of studies surrounding them have increased our understandings of the navigation mechanism of birds in recent years. In this paper, we focus on the studies of the information encoding mechanism of the route in the avian goal-directed behavior. We first summarize and introduce the related studies on the role of the Hp and NCL for goal-directed behavior comprehensively. Furthermore, we review the related cooperative interaction studies about the Hp-NCL local network and other relevant brain regions supporting the goal-directed routing information encoding. Finally, we summarize the current situation and prospect the existing important questions in this field. We hope this paper can spark fresh thinking for the following research on routing information encoding mechanism of birds.

## 1. Introduction

Goal-directed behavior relies on learning the relationship between situations, actions, and their outcomes [1]. As a kind of typical spatial navigation behavior, goal-directed navigation is closely related to the migration, foraging, and homing of the animals, in which the important information in the environment is perceived to support the complex decision-making. Therefore, it is essential for individual survival [2]. In this complex behavior, the brain mechanisms of the basic functions of environmental perception and behavioral decision have been widely studied [3]. In 1948, Tolman put forward the hypothesis of “cognitive map” based on a series of behavioral studies, which suggested that animals can form flexible internal representations of spatial relationships in the environment [4]. After that, the discoveries of a series of spatial navigation function related neurons including place cell [5], grid cell [6], head-direction cell [7], boundary cell [8], and speed cell [9] have provided more and more neural evidence for this hypothesis, improving our understanding of the brain mechanism of spatial navigation.

The motion path from the current place to the goal place is called the route. Routing execution involves the planning and adjustment of the path, which is one of the most important tasks in goal-directed navigation. According to the current leading research, the hippocampus (Hp) region of the brain correlates with the maintenance of memories and decision making in the spatial navigation [10]. Specific hippocampal neurons are highly adapted to process and encode information of the surrounding world [11], making Hp essential for flexible navigation. Existing research shows that the mammals with Hp lesions perform worse in the learning and navigation tasks in the new environment, in which the ability of the optimal routing selection is impaired [12,13]. It indicates that Hp may play an important role in the processing and encoding of the routing-related information. However, we can still conclude that a single brain region (like Hp) is insufficient to finish this complex task independently, just like any other neural processes. A large number of studies show that spatial navigation relies on a local network including multiple brain regions with strong interactions among them [14]. The animals in the navigation process need to estimate the direction and distance of the destination based on the internal metric of space according to the cognitive map constructed in the brain to plan the next action, which requires the Hp to interact with the action-planning systems in other brain regions [15]. This also implies that the route planning in the navigation may involve a widely distributed structural network, in which other spatially-related regions may act as processing nodes. Prefrontal cortex (PFC) is an example, which plays an important role in adaptability, flexible behavior, and executive function [16]. In the brain of the bird, the corresponding analogous brain region is the nidopallium caudolaterale (NCL), which is compared to the avian “prefrontal cortex” [17]. Although individual PFC neurons often contain very little purely spatially-related information [18], more complete contextual signals can be found in the large ensembles recordings. In fact, there is more robust contextual information in PFC ensembles than Hp [19]. These above studies show the important role of Hp and PFC/NCL in spatial navigation and the interaction between them may support the encoding and retrieval of the spatially-related information.

Birds have excellent spatial navigation capabilities. Studies have found that the Hp and NCL regions are thought to be functionally equivalent to mammalian Hp and PFC, sharing similar important roles in spatial cognition [20,21]. For a long time, birds have always been the typical model animal to study the spatial perception and representation mechanism of navigation. Many researchers have tried to propose various hypotheses to explain the complex mechanism [22,23,24,25]. Although we still have different opinions on these hypotheses, it is certain that the spatially-related information in these hypotheses will be projected to the specific brain regions for processing and integration [26]. The results of spatial navigation tasks with damaged brain region have confirmed that Hp, NCL, and their local networks may play important roles in spatial navigation and working memory [27,28], for the information encoding in path planning and adjustment. At present, the studies about goal-directed routing information encoding of the bird mainly focus on the role of a single brain region, while the collaborative relationship and interactions among multiple brain regions, which may play key roles to reveal its neural mechanism further, needs to be studied thoroughly. In this paper, a series of studies related to the role of avian Hp and NCL in goal-directed routing information encoding are briefly reviewed, and we summarize the current situation and prospect the existing problems based on these current studies. It should be noted in particular that we only discuss the Hp and NCL related mechanisms of the routing information coding in spatial cognition and navigation in this review; other mechanisms, such as striatal mediated habit-route encoding mechanism and so on, are outside the scope of this paper.

## 2. The Role of Hp in Goal-Directed Route Information Encoding

Since the place cell was found by O ‘Keefe in the Hp region of rat, which is regarded as the basic unit of the spatial cognitive map [5], Hp has been the most concerned target brain region in the study of spatial navigation mechanism. The place cells in the Hp show place-specific firing activities [11]; the corresponding particular places where the place cell fires in the environment are called place fields [29]. Research has indicated that as the animal traverses the place field of a place cell, the phase at which spikes fire gradually shifts earlier with each theta cycle, a phenomenon called theta precession [30]. Thus, the activities of place cells during theta oscillations seem well suited to encode information about the animal’s current location and a local trajectory on a rapid timescale, which is very important for the spatial decision-making behavior of animals in the environment. An early study has shown that the firing activities of Hp place cells in the maze have nothing to do with the external stimulation itself but encode the intended destination, which suggests that Hp activities are closely related to the future behavior choice of animals [31]. Before the animal starts from any new starting position to a known destination in a goal-directed task, a short sequence of neural activities in Hp place cell network will be generated to encode the spatial motion trajectory to be executed [32]. The further experimental study has shown that the spike sequences of place cells during one theta cycle are temporally compressed representations of an animal’s trajectory [33], reflecting the next routing information. In recent years, a series of studies on rodents have shown that the Hp activities not only represent the current location information under the active state but also can realize the representation of navigational path planning [34], supporting a series of functions including spatial learning, cognitive map forming and maintaining [35]. Besides, studies have shown that the synchronized activities of Hp ensembles under a series of different rhythms including slow gamma (25–50 Hz) and sharp wave ripples (SWRs) (100–250 Hz) also play important roles for the dynamic encoding of routing information [36,37]. After being familiar with a specific route, the Hp cells will replay the activity sequence of the experienced places, which is believed to be helpful for animals to learn and remember the route to the destination [38]. These above studies confirm that Hp plays an important role in spatial navigational routing planning.

**Figure 1:**
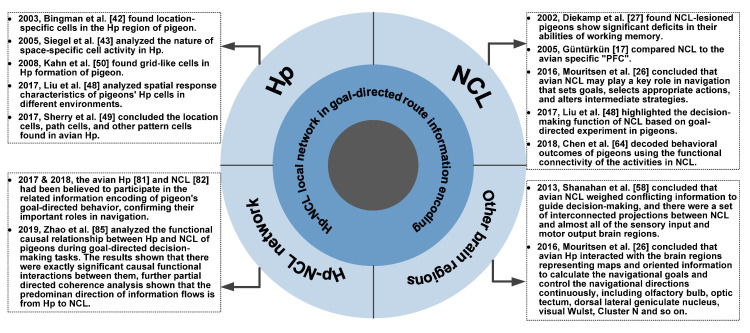
The developments on the role of the avian Hp-NCL local network in goal-directed navigation route information encoding. Hp: hippocampus, NCL: nidopallium caudalle.

The differences between species lead to many differences in encoding characteristics of the Hp between birds and mammals. Research has shown that the structures of avian Hp and mammalian Hp are very different and the correspondence between the subdivisions of them is still debated [39]. However, studies on the functional homology between them have long been concluded that there are similar receptor neurons and functional subregions in their Hp formations [40]. Specifically, some researchers have included that the dorsal avian Hp corresponds to the posterior Hp of primates, which mainly participates in the encoding of spatial memory and contextual memory and performs cognitive functions. The ventral avian Hp corresponds to the dentate gyrus of primates, which is responsible to deal with emotional and social behavior. In fact, studies on spatial memory have shown that the location-specific cells in Hp of birds and mammals also have similar characteristics, and both of them are important for spatial navigation [41]. Compared with rats and other mammals, the studies about avian spatial navigation mechanisms including pigeon started late. Bingman et al., found location-specific cells in the Hp region of pigeons for the first time [42], followed by the research which showed that most of these kinds of cells of pigeons have multiple place fields [43]. These cells share many of the characteristics of those found in mammalian Hp [44], except that they are very unreliable, noisy, and rather broad, whose responses are not as spatially selective as in rats [41]. Also, the related studies on other species of birds such as zebra finch [45,46] and domestic chick [47] also have shown that avian Hp is indeed involved in similar aspects of spatial navigation that are directly comparable to studies with mice. In an experimental study based on various spatial cognitive tasks, the spatial response characteristics of pigeons’ Hp cells in different environments were compared, and the qualitative, quantitative, and stability analysis of their place fields was carried out, in which the results verified the conclusion that Hp cells of pigeon have multiple place fields [48]. Although some experiments have confirmed that there are differences in encoding characteristics of these cells between left and right Hp of pigeons, the spatial encoding ability of Hp has been widely recognized, indicating that the Hp of pigeon participates in many behaviors, such as homing, foraging, and so on. The experiment has shown that specific cells in the Hp region will fire when pigeons reach the spatial goals and pass through the specific path in the maze, and the finding of these spatially responsive neurons in the avian Hp including location cells, path cells, and pattern cells indicates that Hp is very important for avian spatial cognitive learning and route planning [49]. On the other hand, although there is no solid evidence to show the existence of grid cells in the avian Hp, some especial pattern cells have been found. This kind of cells displayed several small, regularly distributed patches with sharp boundaries, whose firing rate maps superficially resemble grid cells recorded from the entorhinal cortex of rats [50]. Although these grid-like cells are not exactly grid cells, they are comparable to rat grid cells to some extent. Also, the results of Hp lesion experiments shown that the Hp-lesioned pigeons lost their abilities to orient their vanishing bearings towards home from a familiar training site following phase shift and anosmic treatment [28], which further confirmed the key role of Hp in avian navigational behavior [51]. Birds have provided the most compelling evidence to indicate that the Hp plays a critical role in spatial memory and cognition, primarily supporting the learning and utilization of map-like, spatial representations of familiar landmarks that can be used to guide navigation over-familiar space [26,52]. We have concluded the representative developments on the role of the avian Hp in goal-directed navigation route information encoding, which is shown in Figure 1. These above studies confirmed the existence of location-specific cells in the avian Hp and also help to explain its important role in spatial information perception and representation to support routing planning in navigation behavior.

## 3. The Role of NCL in Goal-Directed Route Information Encoding

As a complex high-level cognitive process, spatial navigation behavior requires animals to make real-time responses to the changing environmental needs to support flexible decision-making. In fact, PFC acts as a core element that bridges stimulus perception with response execution to ensure adaptive behavior in the distributed cortical network [16]. Since the discovery that mammalian PFC integrates important information for goal-directed navigational behavior in 1987, a large number of studies have confirmed the key role of PFC in spatial processing [53,54]. Early studies have found that PFC cells display more active firing than Hp place cells in the whole environment [11], indicating that individual PFC cells tend to contain very little purely spatially-related information [18]. Further research has shown that the representation of spatial information and behavior correlation is realized at the level of ensembles [19,55]. The results of a Barnes maze-based spatial reference memory task also confirmed that a representation of the behavioral goal emerged in PFC spiking patterns exclusively in the spatial navigation strategy [56].

As a center of higher-order sensory integration, avian NCL has been proposed as a functional analog of the mammalian prefrontal cortex based on hodological, electrophysiological, functional, and neurochemical evidence [17]. The similarities between them are thought to represent an instance of analogy due to parallel evolution [57]. NCL is a convergence zone between ascending sensory and descending motor pathways in which a large number of sensory inputs reach the NCL via a set of interconnected pathways and there are projects to all motor output areas [58], suggesting its key role functionally linking sensory input to motor output. According to the convergence of brain and cognitive evolution in mammals and birds, the avian NCL receives data from all relevant cues and weigh against each other to make a real-time decision, thus making it be compared to the avian specific “PFC” [17]. The results of lesion experiments show that the NCL-lesioned pigeons show significant deficits in their abilities of reversal learning [59] and working memory [27]. Mouritsen et al. reviewed the neural basis of bird navigation in 2016 and concluded that avian NCL may play a key role in navigation as a higher cognitive structure that sets goals, selects appropriate actions, and alters intermediate strategies when new and unexpected information becomes available [26]. In fact, multiscale and multisensory cue integration in the brain is needed for precise navigation [60], which also implies the important role of the avian NCL as a center of higher-order sensory integration and for spatial encoding [61]. The results in the plus-maze based goal-directed experiment in pigeons highlighted the decision-making function of NCL [62]. Further study has indicated that the increased gamma band (40–60 Hz) energy of local field potential (LFP) in NCL is significantly correlated to the goal-directed behavior during the decision-making process [63], and the functional connectivity of the functional network based on the multi-channel spike and LFP activities in NCL can be used for goal-directed behavioral outcomes decoding effectively [64], confirming the role of NCL in the navigational behavior of pigeon on different scales. In addition, for the reward encoding in goal-directed behavior, studies have shown that the activities of NCL were modulated by the value of the reward that would be received based on the reward amount and the delay to reward [65,66], which confirms that NCL has the function of reward encoding to achieve a specific purpose. On the other hand, the NCL neuronal responses during serial-order behavior in pigeons also confirmed that NCL encoded the planning and working memory content containing ordinal knowledge [67,68], which could imply the important role of NCL in the goal-directed navigation, a typical serial-order behavior. The representative developments on the role of the avian NCL in goal-directed navigation route information encoding are concluded in Figure 1. These above studies provide evidence for the central key role of NCL in navigation from a variety of perspectives.

## 4. The Role of Hp-NCL Local Network in Goal-Directed Route Information Encoding

As a complex cognitive process, navigation behavior strongly relies on the ability of the brain to encode the spatial information of the surrounding environment. The involved spatial learning needs to process the real-time input information, which relies on the network composed of multiple brain regions. Their interactions enable animals to complete difficult tasks such as spatial memory, navigation decision-making, and path adjustment [69]. The research on mammals has shown that some neurons projecting to the Hp can receive direct input from PFC [70], which provides a neural basis for the functional connection and interaction between them from the angle of physiological anatomy. An early pioneering study on neural activities in multiple brain regions during working memory showed that the synchronization of Hp-PFC is selectively enhanced during the choice epochs [71]. The brain lesions research found that the inactivation of Hp and PFC would significantly reduce the spatial learning performance of animals [72], and the suppression of the Hp-PFC input signal also impaired the gamma synchronization of the sample epochs and the ability of PFC neurons to encode spatial locations, which have been interpreted as important evidence that Hp-PFC interaction correlates to behavior related spatial encoding [73]. The researchers believe that both Hp and PFC participate in a distributed neural network to interact with each other to realize the real-time dynamic routing planning and navigation, in which PFC cells provide a coarse encoding of goal location, while Hp place cells represent the geometry of the current environment and the identification of a goal location, providing more elaborate encoding for the rat to plan accurate trajectories in space [74,75]. Based on Hp-PFC theta oscillation synchronization in a spatial cognitive task, the Hp-PFC interactions were further explored and the results indicated that the modulation of Hp activity to PFC is helpful to the successful execution of spatial working memory [76]. A series of recent studies have also highlighted the Hp-PFC coordination mechanism in spatial working memory tasks. The analyses on the neural activities of the Hp-PFC circuit have indicated that interregional oscillatory coupling increase with learning during the acquisition of spatial reference memory [56]. The population decoding analyses based on the Hp-PFC ensembles activities during spatial memory-guided behavior have shown that encoding of spatial position is coherent for Hp and PFC ensembles, indicating a theta-oscillation mediated mechanism of temporal coordination for shared processing and communication of spatial information across them [77]. In addition, the coordinated Hp-PFC replays during the learning of a spatial alternation task can support an internal cognitive search of available possibilities for prediction of past and future behavioral paths [78], implying the potential value of Hp-PFC interactions in spatial working memory. The researchers agreed that the Hp-PFC interactions mediated by replay and theta sequences supported memory encoding and retrieval, deliberative decision making, planning, and guiding future actions, playing complementary and overlapping roles at different stages in learning [79].

Compared with the studies of mammalian Hp-PFC interactions during spatial navigation tasks, there are few similar studies on the avian Hp-NCL interactions. Existing studies have shown that both Hp and NCL cells of birds are significantly correlated to their learning and cognitive functions [80]. The rhythm and functional network analyses of the Hp [81] and NCL [64,82] activities in the studies on the encoding mechanism of pigeon’s goal-directed behavior have shown that both of them participate in the related information encoding, confirming their important roles in goal-directed behavior. The researchers believe that Hp of pigeons focuses on the encoding of the current location and goal location, while NCL focuses on the encoding of routing information related to goal-directed behavioral decision-making. They further hypothesize that Hp and NCL of pigeons have a cooperative relationship in goal-directed decision-making tasks since they encode the involved different information. There is no final conclusion about the connection between the avian Hp and NCL now. However, most studies believe that there is a rare direct link between avian NCL and Hp [58,83,84], and clear and solid evidence for the transfer of information from the Hp to the NCL or other extrahippocampal regions is lacking, which makes Hp-NCL network very different from the Hp-PFC network in mammals. A recent study recorded the LFP signals of Hp and NCL respectively when pigeons performed goal-directed decision-making tasks and analyzed the functional causal relationship between them [85]. The results of amplitude–amplitude coupling and LFP functional network analysis shown that there were exactly significant causal functional interactions between Hp and NCL, verifying the above hypothesize. Further results of partial directed coherence analysis show that the predominant direction of information flows is from Hp to NCL, laying a foundation for the future research of avian Hp-NCL interactions in goal-directed navigational behavior. We concluded the representative developments on the role of the avian Hp-NCL network in goal-directed navigation route information encoding in Figure 1. These studies further confirm the important roles of avian Hp and NCL in goal-directed behavior and preliminarily confirmed the existence of interactions between them, implying the potential role of Hp-NCL local network in routing information encoding.

## 5. The Interactions Between Hp-NCL Network and Other Brain Regions

Current studies generally believe that various learning processes, including goal-directed behavior, need to rely on the interactions among the distributed brain networks composed of multiple brain structures to achieve the propose of the behavior control. Routing information encoding in spatial navigation is an important part of goal-directed behavior, in which Hp and PFC/NCL obviously play fundamental but important roles. Even so, they still need to cooperate with other brain regions to be responsible for this complex neural process. Studies on mammals have shown that many other brain regions including visual areas, auditory areas, parietal areas, the amygdala, the striatum, and even the cerebellum are implicated in spatial processing [11]. All of them play specific roles even may serve as more central processing nodes for the neural network analyses involved in spatial cognitive processes. For example, the amygdala plays an important role in spatial processing related to emotional learning and memory [86], and the basal ganglia is involved in the acquisition, storage, and retrieval of spatially related stimulus-reward associations [87]. The striatum seems to be an important node that can transform navigational information into fast adaptive action, considering that its neural responses correlate with the orientation, position, and motor selectivity in spatial navigation [88].

The reliance of birds on navigational ability makes the species able to obtain as much potential relevant oriented information as possible to gain evolutionary advantages. It is necessary for the birds to possess the ability to integrate and weigh all the information related to spatial sensory in the brain [26], which is a process supported by multiple brain regions cooperating with each other [89]. Similar to mammals, the extensive interactions among avian Hp, NCL, and the other brain regions play a key role in supporting the complex task executions in spatial navigation. Avian Hp can interact with the brain regions representing maps and oriented information to calculate the navigational goals and control the navigational directions continuously. These regions include the olfactory area (mainly refers to olfactory bulb), visual area (including optic tectum, dorsal lateral geniculate nucleus, and visual Wulst), and Cluster N, which stores and processes the magnetic compass information [26]. Avian NCL is mainly involved in the integration of all body information and is responsible for weighing the conflicting information to guide decision-making, helping the animal respond to unexpected situations in navigation. As a convergence zone linking the ascending sensory pathway (including trigeminal nerve, visual area, and olfactory area) with the descending motor pathways, there are a set of interconnected projections between NCL and almost all of the sensory input and motor output brain regions [58]. Based on these structural connections, their functional interactions are believed to contribute to the cognition and decision-making performance in goal-directed routing information encoding. The representative developments on the interactions between the Hp-NCL network and other brain regions are shown in Figure 1. Most of the current studies pay more attention to the structural neural projection among the brain regions closely related to navigation including Hp and NCL. However, the functional interactions among them in goal-directed navigational behavior, especially in routing information encoding, needs to be further studied.

## 6. Conclusions and Future Perspectives

**Figure 2:**
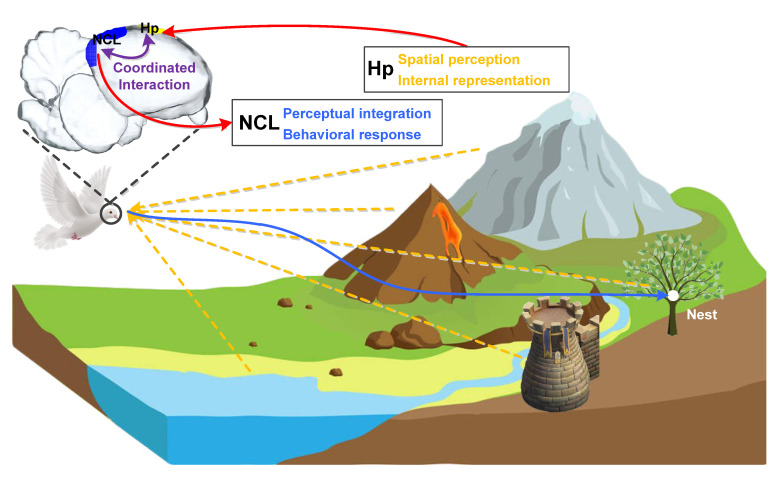
Avian Hp-NCL network performs goal-directed navigation to the nest via coordinated interactions. Hp: hippocampus, NCL: nidopallium caudalle.

After years of research, it is now known that both the avian Hp responsible for spatial perception and internal representation and the NCL responsible for multisensory perceptual integration and behavioral response play important roles in the navigation process. We have more reasons to believe that the realization of this complex behavior must require the cooperation of multiple brain regions including the two. Figure 2 shows the navigation process of a bird with its nest as the goal, in which its Hp and NCL perform different functions by encoding different information in goal-directed navigation and support route planning and adjustment through coordinated interactions. In this process, Hp is mainly responsible for the spatial perception of the environment and the corresponding internal representation in the brain, focusing on the encoding of the current location, goal location, and the geometric characteristics of the environment. While NCL is mainly responsible for linking multisensory perceptual integration with a behavioral response to provide a necessary supplement for the information encoding in Hp, focusing on the encoding of routing information and behavioral choice. As far as the coordinated interactions between them in the goal-directed navigation are concerned, the existing information related to spatial cognition and navigation is perceived and first stored in Hp, and then transmitted to NCL after preliminary processing and encoding. In NCL, these existing information and goal-related information are further integrated, and the next traveling direction will be made after weighing. The reprocessed goal and decision-related information may be transmitted to Hp again to activate the neural activities for further routing information encoding, guiding the goal-directed navigation.

Goal-directed navigation is very important for animals’ daily life and survival. The existing evidence suggest that the studies on the neural mechanism of avian goal-directed routing information encoding will enrich the comparison of spatial navigation mechanisms among different species and improve the theoretical system of navigation research. At the same time, the related studies will promote our understanding of animal spatial navigation mechanism and deepen the research of biologically plausible navigation modeling, hastening the birth of novel intelligent robot technologies. Although the current studies have provided us with a preliminary blueprint of avian goal-directed routing information encoding, many fascinating questions remain unanswered. The nine questions in Table 1 are a summary of the most important mechanistic questions that arose from preparing this review (the order does not indicate relative importance). To answer them, long-term collaborative efforts combining multidisciplinary approaches from neuroscience, biophysics, biochemistry, ethology, and genetics will be required. There is a long way to go but the future can be expected. We should work together to witness these exciting times in the field.

## Figures and Tables

**Table 1:** Important questions in the current research on avian goal-directed navigation.

No.	Description of the Question
1	Do any other mammalian like functional specific cells (such as grid cells) exist in the avian brain to support spatial representation except location cells and some other pattern cells?
2	Are there any navigation-related functional specific cells in avian NCL to support spatial cue integration or navigational behavioral response?
3	How do different types of cells support spatial perception, memory, and decision-making?
4	How is goal-directed spatial perception information processed and transmitted in avian Hp?
5	How is the multisensory navigational information integrated and weighted to guide the decision-making action output in NCL?
6	How do different brain regions coordinated encode the goal-directed navigational-related information in the process of route learning?
7	How can different brain regions interact with each other to dynamically represent the routing information to adjust the path when obstacles are encountered?
8	How do the more widely distributed brain networks cooperate and interact with each other to support goal-directed navigation except avian Hp and NCL?
9	Is the spatial navigational mechanism of the limited space concluded in the laboratory environment consistent with the large-scale mechanism in the natural environment?
